# Towards the targeted management of Chediak-Higashi syndrome

**DOI:** 10.1186/s13023-014-0132-6

**Published:** 2014-08-18

**Authors:** Maria L Lozano, Jose Rivera, Isabel Sánchez-Guiu, Vicente Vicente

**Affiliations:** Centro Regional de Hemodonación, Hospital JM Morales Meseguer, University of Murcia, IMIB-Arrixaca, C/Ronda de Garay s/n, Murcia, 30003 Spain

**Keywords:** Chediak-Higashi syndrome, LYST/CHS1, Phenotype-genotype correlation, CTL cytotoxicity, Hemophagocytic lymphohistiocytosis

## Abstract

Chediak-Higashi syndrome (CHS) is a rare, autosomal recessive congenital immunodeficiency caused by mutations in *CHS1*, a gene encoding a putative lysosomal trafficking protein. In the majority of patients, this disorder is typically characterized by infantile-onset hemophagocytic lymphohistiocytosis (HLH), which is lethal unless allogeneic transplantation is performed. A small number of individuals have the attenuated form of the disease and do not benefit from transplant. Improved outcomes of transplantation have been reported when performed before the development of HLH, thus it is important to quickly differentiate patients that present with the childhood form of disease and to prematurely enroll them into a transplantation protocol. In addition, this would also preclude those that exhibit clinical phenotypes of adolescent and adult CHS from this treatment. Patients with an absence of cytotoxic T lymphocyte (CTL) function have a high risk for developing HLH, and could therefore benefit the most from early hematopoietic stem cell transplantation (HSCT). However, although normal CTL cytotoxicity or bi-allelic missense mutations do not exclude the occurrence of HLH in childhood, a more conservative approach is justified. This article summarizes recent advances in the clinical characterization of CHS patients, provides updates on promising new testing methods, and focuses on specific therapeutic approaches.

## Introduction

Chediak-Higashi syndrome (CHS; MIM #214500; ORPHA167) is a rare autosomal recessive disorder characterized by variable degrees of oculocutaneous albinism, recurrent pyogenic infections, a tendency for mild bleeding, and late neurologic dysfunction. The ‘accelerated phase’ of CHS, namely hemophagocytic lymphohistiocytosis (HLH), develops in 50–85% of patients, and is fatal if not treated. This disorder was first reported by Beguez Cesar, a Cuban pediatrician, in 1943 [1]. Further reports by Chediak [2] and Higashi [3] emphasized the hematologic features of the disease and motivated Sato [4] to associate their names with the anomaly. The occurrence of this disorder is rare, and less than 500 cases have been reported worldwide in the past 20 years [5]. The genetic defect resulting in CHS was identified in 1996 [6,7], and was mapped to human chromosome 1q42–44. The human gene, *CHS1*, was originally called *LYST* for lysosomal trafficking regulator gene (*LYST*, OMIM #606897). It is 87.9% homologous with *Lyst*, the murine gene for beige, a mouse model of human CHS [6,8]. The gene contains 53 exons (51 coding) with an open reading frame of 11,406 bp, and encodes for a 3801 amino acid protein, CHS1. The CHS1 protein is a highly conserved, large cytosolic protein of approximately 430 kDa. The N-terminal extreme contains several ARM/HEAT α-helix repeats, followed by a BEACH domain and a C-terminal domain of seven WD40 repeats. While the exact function of the CHS1 is still unknown, it is thought to play a role in regulating lysosome-related organelle size, fission, and secretion [9]. In this disorder, giant cytoplasmic organelles, such as inclusion bodies, lysosomes, or melanosomes are present in virtually all granulated cells. Furthermore, the exocytic pathway of secretory lysosomes is defective and plasma membrane repair mechanisms are impaired [10]. The underlying biochemical defect in CHS/beige has not been determined, but studies suggest that abnormal down-regulation of protein kinase C (PKC) is responsible for the immune dysfunction [11], and for giant granule formation [12]. Enhanced ceramide production induces calpain-mediated proteolysis of PKC. The ceramide-induced down-regulation of PKC results in abnormal cellular phenotypes, suggesting these metabolic cascades are responsible for the beige phenotype [13].

### Clinical course and diagnosis

Most patients are diagnosed during the first decade of life, and while the disease affects multiple organs and systems, death often occurs early because of infection, bleeding, or development of HLH. Patients with CHS frequently exhibit hypopigmentation, enhanced susceptibility to bruising, recurrent infections, and peripheral neuropathy. The degree of hypopigmentation varies, and typically affects skin, hair, and eyes. A speckled hyperpigmentation or dark skin may uncommonly be seen in more pigmented races, leading to the suspicion of other diseases with a consequent delay in diagnosis [14,15]. Hair color may be blond, gray, or white, often with a distinguished silvery or metallic sheen. Iris hypopigmentation may be associated with decreased retinal pigmentation, and ocular manifestations include photophobia, decreased visual acuity, nystagmus, and strabismus.

Patients are affected by frequent and severe pyogenic infections secondary to the abnormal functions of polymorphonuclear leukocytes. Most children with CHS receive early attention because of troublesome recurrent bacterial infections. The most common sites of infection are the skin, respiratory tract, and mucous membranes. *Staphylococcus* and *Streptococcus* are the species most frequently isolated from these sites. Periodontal disease and bone loss of dental alveoli associated with various microorganisms are common [16]. Patients also show a mild bleeding tendency often manifesting as bruising and mucosal bleeding as a result of defective platelets; however, this does not usually require treatment.

CHS may present with neurologic dysfunction and should be considered in the differential diagnosis of children and young adults first seen with symptoms of spinocerebellar degeneration or movement disorders. Common physical findings include motor and sensory neuropathies, ataxia, tremors, cranial nerve palsies, low cognitive abilities, learning disabilities, and seizures. Patients who survive to the second or third decade may exhibit neurologic deterioration, including parkinsonism and dementia, and are often confined to a wheelchair.

The ‘accelerated phase’ is the most life-threatening clinical feature of CHS, affecting about 85% of CHS patients within the first decade. This manifestation defines the characteristic ‘childhood’ form of the disease and is characterized by massive HLH. It often occurs following initial exposure to Epstein-Barr virus (EBV), when it may resemble lymphoma [17]. HLH manifests as fever, lymphadenopathy, and hepatosplenomegaly with signs of liver dysfunction, cytopenia, and bleeding. Massive lymphohistiocytic infiltration of virtually all organ systems may also be observed. Most patients with prior history suggestive of CHS undergo a variable period of recurrent infections before entering the accelerated phase, but primary presentation in the accelerated phase has also been reported [17-20].

However, about 10–15% of patients follow a less severe clinical course of CHS, the ‘adolescent’ and ‘adult’ forms. These children present with mostly subtle hypopigmentation, a lower frequency of infections during childhood, adolescence, and adulthood, mild bleeding manifestations, and survive until adulthood without experiencing an ‘accelerated phase’. Nonetheless, during adolescence or adulthood they develop progressive neurologic symptoms including intellectual deficit, dementia, peripheral neuropathy, parkinsonism, balance abnormalities, and tremor.

**Figure 1:**
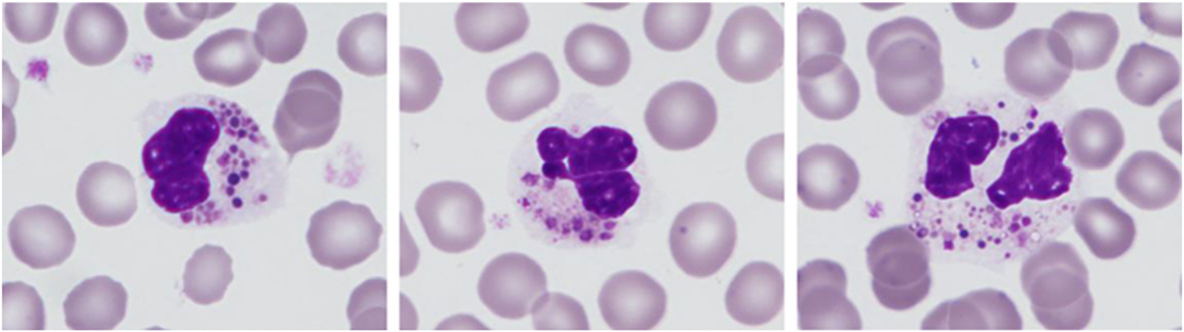
**Wright Giemsa staining of a peripheral blood smear from a patient with Chediak-Higashi syndrome showing polymorphonuclear leukocytes with abundant giant intracytoplasmic granules.**

The diagnosis of CHS is usually established when a child presents to a hospital with partial oculocutaneous albinism and recurrent pyogenic infections, and although unusual, as previously stated, primary presentation in the accelerated phase may also occur. Clinical suspicion is confirmed by laboratory evaluation, imaging studies, and by histologic findings. Indeed, the diagnosis of CHS patients is often made because of incidental observations of giant granules in neutrophils, derived from the coalescence of azurophilic and secondary granules on peripheral blood smears (Figure 1). Giant granules are also observed in lymphocytes and natural killer (NK) cells from patients with CHS. Bone marrow aspirates demonstrate numerous large azurophilic or eosinophilic cytoplasmic inclusion bodies in cells of myeloid lineage that react strongly to peroxidase staining. Ultra-structural studies show that the granules contain giant lysosomes and fibrillary structures in myeloid cells, with a reduced number and irregular morphology of platelet-dense bodies [18].

Microscopic examination of the hair can also reveal clumped melanin granules, larger than those seen in normal hairs, and examination of the skin shows giant melanosomes both in keratinocytes and melanocytes, which can be used as a laboratory test for differential diagnosis with other partial albinism disorders [21]. Murine models of CHS exhibit the neuronal accumulation of giant lysosomes and intra-cytoplasmic inclusions in Purkinje cells of the cerebellum and motor cortex [22].

CHS patients have a profound defect in the function of cytotoxic and NK cells [23]. In addition, defects of neutrophils [24] include ineffective granulopoiesis, moderate neutropenia, and delayed and incomplete degranulation associated with phagocytic, chemotactic, and bacterial killing defects. Platelets are also functionally defective with reduced dense granules and impaired functions. Platelet function studies are consistent with a storage pool deficiency with reduced dense bodies and consequent defects of secretion-dependent aggregation [18,25]. Immunoglobulin levels and complement are generally normal.

Computed tomography scans and magnetic resonance imaging might show diffuse atrophy of the brain and spinal cord [26], while electromyography and electroencephalography might reveal delayed nerve conduction time and seizure activity, respectively. Definite diagnosis is based on the molecular genetic testing of *CHS1*. Prenatal diagnosis is confirmed by genetic testing in chorionic villus cells, amniotic fluid cells, or fetal blood leukocytes.

It is important to establish a differential diagnosis of CHS with other hypopigmentation disorders that are associated with immunodeficiency (Griscelli syndrome type 2, Hermansky-Pudlak syndrome type 2 and type 9, and MAPBPIP deficiency syndrome) [21]. These are also heterogeneous autosomal recessive conditions caused by defects in genes that encode proteins with specific functional roles in secretory lysosomes, which also manifest as hypopigmentation, impaired primary hemostasis, decreased blood cell counts, and lymphocyte cytotoxic activity against microbial pathogens. While differential diagnosis requires biochemical and molecular criteria, clinical manifestations that direct diagnosis towards CHS include ocular symptoms, such as nystagmus, neurologic disease, transient neutropenia, and HLH. The presence of giant inclusions by the microscopic examination of peripheral blood is typical and exclusive to this disorder.

### Hemophagocytic lymphohistiocytosis

Hemophagocytic lymphohistiocytosis, also termed the ‘accelerated phase’, is a hyperinflammatory syndrome with a high mortality rate, even in those receiving appropriate treatment, which affects more than 75% of CHS patients within their first decade. The basic pathophysiologic mechanism underlying HLH is inappropriate cytotoxic activity [27], leading to impaired down-regulation of immune responses and the sustained activation and proliferation of cytotoxic T lymphocytes (CTLs) and NK cells [21,28]. Activated lymphocytes and macrophages secrete high levels of pro- and anti-inflammatory cytokines and chemokines, giving rise to the characteristic clinical and laboratory findings. Histopathology reveals lymphoproliferative infiltration of the bone marrow and reticuloendothelial system. However, although bone marrow aspirate analysis is commonly used for diagnosis of HLH, it has a sensitivity of only 60% [29].

The diagnosis of HLH must be based on clinical and laboratory criteria, according to the guidelines of the Hystiocyte Society last revised in 2004 [30], and either a genetic defect, such as mutations in *CHS1*, or five of the eight criteria must be met. These include (a) fever, (b) splenomegaly, (c) cytopenias affecting at least two or three cell lineages, (d) hypertriglyceridemia and/or hypofibrinogenemia, (e) hemophagocytosis, (f) low/absent NK-cell activity, (g) hyperferritinemia, and (h) high soluble interleukin-2-receptor (soluble CD25) levels [28]. It must be emphasized that mutations in *CHS1* or a diagnosis of CHS do not prove the acute clinical syndrome of HLH, but rather a predisposition to develop the condition. Additionally, there are several characteristics that may underscore the clinical suspicion: moderate lymph node enlargement, jaundice, edema, pleural or pericardial effusions, skin rash, hypoproteinemia and hyponatremia, and elevation of liver transaminases and lactate dehydrogenase [31]. Although not currently cited in any formal diagnostic algorithm, flow cytometry has been added to the arsenal of screening tools for HLH. The absence or decreased intensity of CD107a measured on the cell surface after degranulation has a high sensitivity and specificity for the diagnosis of a primary (genetic) disorder of granule exocytosis, as opposed to secondary causes of disease [23].

### Predictors of HLH

Allogeneic transplantation from an HLA-matched sibling or from an unrelated donor or cord blood transplant is the treatment of choice to correct the immunologic and hematologic manifestations of early-onset CHS [26]. Transplantation appears to be most successful when performed prior to the accelerated phase [32]. Therefore, it would be of great value to differentiate patients that present with the childhood form of the disease from those that exhibit clinical phenotypes of adolescent and adult CHS so as to prematurely enroll only the former ones into a transplantation protocol.

While most CHS patients will eventually develop HLH, defining the *a priori* risk of an individual patient to develop HLH is still speculative. This may depend not only on the nature of the mutation (null or hypomorphic) that determines the residual lytic activity of CTLs, but also on the time point and nature of exposure to predominantly infectious triggers, and other host factors that can elicit HLH in predisposed individuals. However, recent research has led to a greater understanding of the key variables that could have prognostic influence on the development of this highly aggressive syndrome.

#### Genotype-Phenotype correlation

To date, 63 CHS1/LYST mutations have been described [6,7,14,18,27,33-49], including 31 substitutions (20 nonsense, 11 missense), 19 deletions, 9 insertions, and 4 acceptor splice sites (Table 1). A reasonably straightforward genotype-phenotype correlation of the disease has been suggested: early reports indicate frameshift, nonsense, and splice site mutations resulting in an absent CHS1/LYST protein correlate with severe childhood CHS, whereas milder adolescent or adult forms of CHS present with at least one missense mutation probably encoding a partially functioning protein [5,34,39]. However, this linkage was not observed in other studies [14,27,35,45], and few exceptions are known. Among patients with the severe childhood form of CHS in whom molecular studies have been reported, no bi-allelic missense mutations were detected, except for one case with a molecular change that predicted alterations in both the structure and electrostatic surface potential of the protein [18]. However, several bi-allelic truncating mutations have been observed in patients with the adolescent or adult form of the disease (Table 1). Thus, while homozygous or bi-allelic missense mutations are usually associated with later-onset CHS, protein truncating mutations have been described both in patients with the childhood and adult forms of the disease. As a consequence, in individuals with homozygous truncating mutations, the time of onset of HLH seems to be determined both genetically and by environmental factors (see below), while attempts to link genotypes and clinical phenotypes in patients carrying bi-allelic missense mutations ideally require the identification of the actual molecular effect of the molecular change.

**Table 1:** ***CHS1*** **mutations, illness severity, and effect on CHS1 protein of patients described in the literature**

**‘Childhood’ form of the disease**					**‘Adolescent’or ‘Adult’ form of the disease**				
**Genotype**	**Effect on LYST mRNA**	**mRNA**	**Protein**	**Reference**	**Genotype**	**Effect on LYST mRNA**	**mRNA**	**Protein**	**Reference**
Homozygous	Nonsense/frameshift	c.1467delG	E489fsX566	[7]	Homozygous	Nonsense/frameshift	c.5784 + 5G > T	Acceptor splice site	[27]
Homozygous	Nonsense/frameshift	c.1899insA	K633fsX638	[33]	Homozygous	Missense	c.5996 T > A	V1999D	[34]
Homozygous	Nonsense/frameshift	c.9590delA	Y3197fsX3258	[33,35,36]	Homozygous	Missense	c.9827_9832del6bp	N3276_T3277del	[37]
Homozygous	Nonsense/frameshift	c.3085C > T	Q1029X	[38]	Compound heterozygous	Missense	c.10127A > G	N3376S	[39]
						Nonsense/frameshift	c.2413delG	E805fsX806	
Homozygous	Nonsense/frameshift	c.2620delT	F874fsX898	[35,40]	Compound heterozygous	Missense	c.8428G > A	E2810K	[34]
						Nonsense/frameshift	c.4274delT	L1425fsX1426	
Homozygous	Nonsense/frameshift	c.10395delA	K3465fsX3467	[34]	Compound heterozygous	Missense	c.4361C > A	A1454D	[34]
						Nonsense/frameshift	c.5061 T > A	Y1687X	
Homozygous	Nonsense/frameshift	c.7060-7066del 7 bp	L2354fsX2369	[36]	Compound heterozygous	Missense	c.9925G > A	G3309S	[41]
						Nonsense/frameshift	c.1507C > T	R503X	
Homozygous	Nonsense/frameshift	c.7555delT	Y2519fsX2528	[35]	Heterozygous	Nonsense/frameshift	c.8583G > A	W2861X	[34]
Homozygous	Nonsense/frameshift	c.9106-9161del 56 bp	G3036fsX3051	[35]	Heterozygous	Nonsense/frameshift	c.148C > T	R50X	[38]
Homozygous	Nonsense/frameshift	c.6078C > A	Y2026X	[34]	Heterozygous	Nonsense/frameshift	c.3944-3945insC	Q1847fsX1850	[42]
Homozygous	Nonsense/frameshift	c.5004delA	G1668fsX1717	[36]	Homozygous	Nonsense/frameshift	c.3310C > T	R1104X	[7]
Homozygous	Nonsense/frameshift	c.5519delC	S1840fsX1842	[36]	Homozygous	Nonsense/frameshift	c.575insT	L192FfsX6	[36]
Homozygous	Nonsense/frameshift	c.3310C > T	R1104X	[35]	Homozygous	Nonsense/frameshift	c.575_576insT	L192fsX197	[27]
Homozygous	Nonsense/frameshift	c.11102G > T	E3668X	[43]	Homozygous	Nonsense/frameshift	c.3310C > T	R1104X	[27]
Homozygous	Nonsense/frameshift	c.5506C > T	R1836X	[14]	Homozygous	Missense	c.961 T > C	C258R	[18]
Homozygous	Nonsense/frameshift	c.7060-1G > A	Acceptor splice site	[27]	Homozygous	Missense	c.4189 T > G	F1397V	[44]
Homozygous	Nonsense/frameshift	c.10551_10552del2	Y3517X	[27]	Homozygous	Missense	c.4688G > A	R1563H	[34]
Homozygous	Nonsense/frameshift	c.5506C > T	R1836X	[27]	Homozygous				
Homozygous	Nonsense/frameshift	c.2374_2375delGA	D792fsX797	[27]					
Homozygous	Nonsense/frameshift	c.4508C > G	S1483X	[27]					
Homozygous	Nonsense/frameshift	c.5506C > T	R1836X	[27]					
Compound	Missense	c.2570C > G	S857C	[27]					
heterozygous	Nonsense/frameshift	c.9930delT	F3310fsX3346						
Compound heterozygous	Nonsense/frameshift	c.1540C > T	R514X	[45]					
		c.9893delT	F3298fsX3304						
Compound heterozygous	Nonsense/frameshift	c.3622C > T	Q1208X	[46]					
		c.11002G > T	E3668X						
Compound heterozygous	Nonsense/frameshift	c.10445insCA	V3483fsX3516	[47]					
		Not specified	R2403X						
Heterozygous	Nonsense/frameshift	c.5317delA	R1773fsX1785	[35]					
Heterozygous	Nonsense/frameshift	c.9228ins 10 bp	K3077fsX3080	[35]					
Heterozygous	Nonsense/frameshift	c.118insG	A40fsX63	[6]					
Heterozygous	Nonsense/frameshift	c.3073 + 3074delA	N1025fsX1030	[38]					
Heterozygous	Nonsense/frameshift	c.2454delA	K818fsX823	[34]					
Heterozygous	Nonsense/frameshift	c.3434-3435insA	H1145fsX1153	[34]					
Heterozygous	Nonsense/frameshift	c.4052C > G	S1351X	[34]					
Heterozygous	Nonsense/frameshift	c.3944-3945insC	T1315fsX1331	[42]					
Compound heterozygous	Nonsense/frameshift	c.7060-1G > T	Acceptor splice sites	[27]					
		c.11196-1G > A							
Homozygous	Missense	c.11362G > A	G3725R	[18]					
Homozygous	Nonsense/frameshift	c.925C > T	R309X	[48]					

The type of presentation is unknown in two patients: (1) compound heterozygosity for c.7982C > G and c.8281A > T frameshift mutations [42]; (2) c.10747G > homozygous missense mutation [49]. CHS, Chediak-Higashi syndrome. GenBank: U67615.1.

#### Differences in CTL cytotoxicity

In a normal physiologic context, CTLs are required for the clearance of viral infection as well as the regulation and termination of inflammatory responses. The relevance of immune surveillance performed by CTLs when inherited deficiencies, such as CHS, impair cellular cytotoxic activity is emphasized by the absence of virus control leading to sustained polyclonal CTL activation, and subsequent excessive macrophage activation characteristic of HLH [50].

In this context, the comparative analysis of CTL function in CHS patients demonstrated a significant correlation between the degree of lytic impairment in CTLs and disease phenotype, suggesting this “immunophenotype”-phenotype correlation might be helpful for clinical decisions [27,51]. Thus, CTL cytotoxicity has been shown to be reduced in CHS patients with early-onset HLH, whereas it was retained in patients who later or never developed HLH. This suggests that subtle differences in CTL function can determine whether an infection triggers full HLH or is eliminated without any significant manifestations of disease. An animal model of CHS (*souris*) also demonstrated that graded virus load in the spleen after infection, related to the degree of impaired CTL cytotoxicity, paralleled HLH severity [52]. Jessen *et al.* [27] suggested that patients with absent CTL cytotoxicity show an indication for early allogeneic hematopoietic stem cell transplantation (HSCT) because of their high risk of developing HLH. While cytotoxicity and virus control appear to be key variables in the phenotype of patients, further investigation is expected to confirm such findings, and to point to additional treatment approaches to avoid this highly aggressive syndrome.

#### Cellular defects

Platelets from patients presenting with “childhood” CHS have been reported to be markedly deficient or lacking in dense bodies [25,53], while platelet granules are often present in platelets from patients with late-onset CHS [18,39]. This observation is also supported by the finding that platelets from individuals with the milder form of disease exhibit a reduced impairment of platelet function compared with patients presenting with the severe early-onset disease [18,54]. Other than platelets, the cellular characteristics of lysosome-related organelles of various cell types derived from patients with different forms of CHS may also correlate with the clinical phenotype and molecular phenotype [39].

### Treatment

In general, the backbone of treatment for CHS focuses on three main areas: supportive management of disease-derived complications, treatment of the “accelerated phase” or HLH, and HSCT. A prompt diagnosis of CHS, with an emphasis on the molecular characterization and analysis of CTL cytotoxicity, can help identify patients with a high risk of developing HLH. The most effective treatment for hematologic and immune defects is HSCT, albeit there is no evidence of efficacy in delaying or preventing progressive neurologic dysfunction [5,32,55].

#### Supportive management of disease-derived complications

The management of patients with CHS begins with early disease identification and diagnosis. Families with affected children require counseling regarding the risk of the same disorder occurring in future children. While the parents of an affected child are usually obligate heterozygotes and therefore asymptomatic, two cases of CHS caused by uniparental disomy of chromosome 1 have been reported [40,43].

Measures to prevent routine infections include education of the child and caregivers regarding effective hygiene, and meticulous attention to oral and dental care. Skin protection and sunglasses should be used to prevent sunburn and to protect sensitive eyes from ultraviolet light. While these patients can safely receive all killed or inactivated vaccines, live vaccines are contraindicated. The duration of antimicrobial therapy to treat common infections should ideally be two to three times longer than standard recommendations [56]. Granulocyte colony-stimulating factor (G-CSF) can be used to improve or correct neutropenia and decrease infections [57]. Administration of a potent calpain inhibitor, E-64-d, which protects PKC from proteolysis, decreases the susceptibility to *Staphylococcus aureus* infection in a mouse model of CHS (beige mice) [58], and improves NK and bactericidal activity in cells isolated from CHS patients *in vitro* [59]. These data suggest that correction of the abnormal down-regulation of PKC in CHS by specific treatments may be an effective treatment to counteract infections. Nevertheless, apart from preclinical studies, including both *in vitro* and animal assays, no information on the safety and efficacy of these drugs in CHS patients is available.

Patients may exhibit an increased bleeding tendency owing to platelet dysfunction caused by delta storage pool deficiency. Preventative measures include avoidance of drugs that interfere with platelet functions such as aspirin, other non-steroidal anti-inflammatory agents, or serotonin reuptake inhibitors. Intramuscular injections are prohibited, but subcutaneous injections are authorized [25]. Careful dental hygiene can minimize gingival bleeding and treatment with desmopressin and/or anti-fibrinolytic agents is effective in preventing bleeding after dental extraction or minor surgery in patients with storage pool disease or mild bleeding disorders [60,61]. Platelet transfusions are particularly indicated in cases of severe uncontrolled bleeding, when prior treatments have been unsuccessful, and/or in the presence of, or anticipation of, excessive traumatic or surgical bleeding.

Data on pregnancy in female CHS patients are very scarce, suggesting that gestation does not seem to exert any influence on the course of disease in the affected mother, and that no specific measures concerning pregnancy, labor, or delivery have to be taken [62]. Furthermore, two male patients with an attenuated form of the disease fathered children despite below normal levels of testosterone [37].

It has been suggested that neurologic manifestations result directly from defective CHS1 in neurons and glial cells, or from lymphocytic tissue infiltration during the accelerated phase of disease [37]. Furthermore, neurologic disease was shown to develop despite successful HSCT [26]. A subset of CHS patients presenting with the adult form of the disorder have a muted pigmentary or hematologic presentation while their neurologic symptoms dominate the disease. Patients with this pattern of manifestations might benefit, at least in the short term, from L-dopa, selegiline, trihexylphenidyl, biperiden, or amantadine treatment [41,63-65]. Additionally, ophthalmologists should be aware that progressive visual loss and constriction of the visual field can occur in patients with CHS as they grow older [66]. At diagnosis, patients would benefit from a multidisciplinary input from a neurologist with expertise in movement disorders, as well as a clinical immunologist (even if historic clues suggesting immunodeficiency are absent), an ophthalmologist, a neurophysiologist, and a neuroradiologist [37].

Even though there are no general recommendations for clinical follow-up of patients with adult-onset CHS, regular screening might include physical examination and/or abdominal ultrasound to monitor for hepatosplenomegaly, blood counts to evaluate cytopenias, biochemical tests to assess for signs of liver dysfunction, including serum ferritin levels, hypertriglyceridemia, and/or hypofibrinogenemia.

#### Treatment of the accelerated phase or HLH

The therapy of HLH involves a two-pronged approach aiming to suppress the exaggerated immune response through the use of immunosuppressive agents and a long-term strategy attempting to definitively correct the underlying genetic defect by allogeneic HSCT as early as possible, when an acceptable donor is available. The HLH-94 protocol includes dexamethasone, etoposide, and intrathecal methotrexate [67], while the 2004 treatment protocol recommends an 8-week induction therapy with corticosteroids, etoposide, and cyclosporine A [30]. In the latter schedule, intrathecal therapy with methotrexate and prednisone is restricted to patients with evidence of central nervous system disease progression after 2 weeks of systemic treatment, or in those with worsening or unimproved cerebrospinal fluid pleocytosis. In patients with CHS and EBV-associated HLH, the addition of rituximab has been reported to be a valuable adjunct to therapy [47], although in contrast to normal EBV infection, in HLH patients, the virus is also present in T cells [68].

Other treatment options include the anti-CD52 monoclonal antibody alemtuzumab as a second-line therapy for pre-transplantation treatment of HLH refractory to etoposide-based treatments [69]. In addition, based on previous data [70], anti-thymocyte globulin has been incorporated into a novel hybrid therapy with chemotherapy for the treatment of HLH in a phase II clinical trial, which is now open and enrolling patients (NCT01104025).

Maximal initial supportive care is recommended, and appropriate broad-spectrum antibiotics should be used presumptively until the culture results are available. Supportive therapy includes prophylactic cotrimoxazole, an oral antimycotic, during the initial dexamethasone therapy, consideration of antiviral therapy in patients with ongoing viral infections, and intravenous immunoglobulins (0.5 g/kg) once every 4 weeks (during initial and continuation therapy) [71].

Gastroprotection with ranitidine or other gastroprotective agents is also recommended. G-CSF and blood transfusion should be used as required. Thrombocytopenia, coagulopathy, and DIC may require platelet transfusion, fresh frozen plasma, cryoprecipitates, and fibrinogen. Although there is still limited evidence, recent studies suggest the use of recombinant human thrombopoietin might help reduce severe bleeding and the frequency of platelet transfusion [72]. Furthermore, thrombomodulin may correct coagulation abnormalities associated with disseminated intravascular coagulation [73]. Many patients need to be admitted to the intensive care unit because of requirements of hemodynamic and respiratory support.

Even though the therapeutic results of HLH-2004 in familial HLH, such as CHS, have not been yet published, those of the previous HLH-94 protocol indicate the prognosis without treatment is poor, with a median survival of 1–2 months [66]. Treatment with HSCT improves the 3-year survival of familial HLH, such as CHS, from nearly 0% to 50% [74]. Thus, the only definite long-term therapy of CHS patients surviving HLH, even cases in the accelerated phase, remains allogeneic HSCT [75].

#### Hematopoietic stem cell transplantation

Whereas early-onset CHS is potentially fatal unless the patient undergoes allogeneic HSCT, in juvenile and adult CHS the disability shifts from hematologic dysfunction to predominantly central nervous system deficits. Allogeneic HSCT appears to be the most successful treatment, if performed prior to the accelerated phase in the early-onset form of CHS, for prevention of life-threatening infections and HLH. Thus, using predictors of HLH development might help identify patients with the ‘childhood’ form of the disease who would benefit from HSCT, from those with the adolescent or adult forms, in whom transplantation is precluded. Patients with absent CTL cytotoxicity might have an indication for early HSCT because of their high risk of developing HLH [27]. Normal CTL cytotoxicity or bi-allelic missense mutations do not exclude the occurrence of HLH in childhood, but a more conservative approach seems to be justified [18,27]. No recommendations can be made regarding bi-allelic nonsense/frameshift mutations, because acquired factors, such as viral infections, may determine the triggering of HLH.

Transplantation using matched related or unrelated donors can be curative for the hematologic and immunologic manifestations of CHS and HLH, although it does not affect progressive neurologic deterioration or oculocutaneous albinism [26,32,51,54]. A retrospective review of 35 cases of HSCT in CHS patients indicated a 5-year overall survival of 62%. Patients with active HLH at transplantation exhibited a mortality rate of 58% at analysis, and results were better when the transplant was performed prior to acceleration, or for those with HLH, after response to chemotherapy [32]. Although survival after alternative related donor transplantation (not HLA-identical sibling, including haploidentical donor) was poor in that study, this may be explained by the presence of symptoms of the accelerated phase at transplantation in most recipients. The Histiocytosis Society reported an inferior outcome after conventional myeloablative conditioning (MAC) with a mismatched donor, having a 3-year overall survival of 54% and 50% with a mismatched unrelated and a family haploidentical donor, respectively, compared with 71% and 70% with a matched related and unrelated donor, respectively [74]. However, in a study published a year later, by combining compatibility and disease control status, no significant impact of donor compatibility on survival after HSCT was found [76]. In general, even though the use of matched (related or unrelated) donors might be associated with more favorable survival, alternative donors are acceptable if matched donors are unavailable, because the outcomes are still appropriate.

HSCT with MAC using busulfan and cyclophosphamide with or without etoposide has long been the standard of care for patients with CHS [32,77], but increased mortality has prompted the use of less toxic approaches. Recently, reduced-intensity conditioning (RIC) with fludarabin, melphalan, and alemtuzumab was shown to be associated with lower toxicity and increased survival of patients with primary or familial forms of HLH (RIC 3-year OS of 92% vs. 43% with MAC, P = 0.0001) [78,79]. Mixed chimerism is more frequent after RIC conditioning; however, clinical observations suggest that stable chimerism with 15% donor cells may be sufficient [32]. While the use of cord blood HSCT in HLH has often proved problematic, RIC has also been used in an unrelated cord blood setting with promising results, and engraftment rates of 80% [80].

## Conclusions

Chediak-Higashi syndrome is a rare, likely under-diagnosed, autosomal recessive disorder that affects many organs. The majority of cases (50–85%) have the ‘childhood’ form of the disease, which is universally fatal without treatment, and should be suspected in a child who has partial albinism and a history of recurrent or severe infections. A small percentage of people with CHS have a milder form of the condition, which should be considered when young adults develop neurodegenerative disease in association with pigmentary abnormalities. A timely diagnosis is imperative, and in both forms of disease, the disorder can be easily screened for with a simple, quick, and non-invasive careful examination of a peripheral blood smear. Prognosis is poor in the childhood form of the disease as death frequently occurs in the first decade of life due to infections or the development of HLH. A prompt diagnosis of the early-onset form of disease, with special emphasis on the molecular characterization and analysis of CTL cytotoxicity, might help identify patients with a high risk of developing HLH. Patients with absent CTL cytotoxicity might have an indication for early HSCT because of their high risk of developing HLH. Normal CTL cytotoxicity or bi-allelic missense mutations do not exclude the occurrence of HLH in childhood, but a more conservative approach is justified. Patients with the ‘childhood’ form of the disease that develop HLH before transplantation should receive corticosteroids and etoposide-based regimens prior to inclusion in a transplantation protocol. The most effective treatment for the hematologic and immune defects in patients is HSCT, although there is no evidence of efficacy in delaying or preventing progressive neurologic dysfunction. Reduced intensity conditioning regimens may be better than the traditional myeloablative regimens in terms of toxicity as well as OS, although comparative trials are lacking. Increased awareness and the early identification of patients with the potentially lethal form of the disorder convey unique therapeutic and prognostic implications that may improve outcomes. With a high degree of clinical suspicion, these patients should be immediately referred to a tertiary care center and treated by multidisciplinary teams including hematologists, pediatricians, dermatologists, biologists, neurologists, clinical immunologists, and social workers. Early treatment of children with CHS is of paramount importance.
